# Fine-tuning mitochondrial activity in *Yarrowia lipolytica* for citrate overproduction

**DOI:** 10.1038/s41598-020-79577-4

**Published:** 2021-01-13

**Authors:** Jorgelindo da Veiga Moreira, Mario Jolicoeur, Laurent Schwartz, Sabine Peres

**Affiliations:** 1 Research Laboratory in Applied Metabolic Engineering, Department of Chemical Engineering, Ecole Polytechnique de Montréal, Centre-Ville Station, P.O. Box 6079, Montréal, QC Canada; 2 LRI, Université Paris-Saclay, CNRS, 91405 Orsay, France; 3 MaIAGE, INRAE, Université Paris-Saclay, 78350 Jouy-en-Josas, France; 4 Assistance Publique des Hôpitaux de Paris, 149 avenue Victoria, 75004 Paris, France

**Keywords:** Dynamical systems, Industrial microbiology

## Abstract

*Yarrowia lipolytica* is a non-conventional yeast with promising industrial potentials for lipids and citrate production. It is also widely used for studying mitochondrial respiration due to a respiratory chain like those of mammalian cells. In this study we used a genome-scale model (GEM) of *Y. lipolytica* metabolism and performed a dynamic Flux Balance Analysis (dFBA) algorithm to analyze and identify metabolic levers associated with citrate optimization. Analysis of fluxes at stationary growth phase showed that carbon flux derived from glucose is rewired to citric acid production and lipid accumulation, whereas the oxidative phosphorylation (OxPhos) shifted to the alternative respiration mode through alternative oxidase (AOX) protein. Simulations of optimized citrate secretion flux resulted in a pronounced lipid oxidation along with reactive oxygen species (ROS) generation and AOX flux inhibition. Then, we experimentally challenged AOX inhibition by adding *n*-Propyl Gallate (nPG), a specific AOX inhibitor, on *Y. lipolytica* batch cultures at stationary phase. Our results showed a twofold overproduction of citrate (20.5 g/L) when nPG is added compared to 10.9 g/L under control condition (no nPG addition). These results suggest that ROS management, especially through AOX activity, has a pivotal role on citrate/lipid flux balance in *Y. lipolytica*. All taken together, we thus provide for the first time, a key for the understanding of a predominant metabolic mechanism favoring citrate overproduction in *Y. lipolytica* at the expense of lipids accumulation.

## Introduction

*Yarrowia lipolytica* is a non-conventional yeast usually isolated from dairy products, oil and water environments^1,2^. This oleaginous yeast is emerging as an important cell factory for lipid production^3,4^. Indeed, over 50–70% of its dry cell weight accounts for lipid accumulation^5,6^, and even 90% of its biomass as lipids has been obtain for an engineered *Y. lipolytica* strain^7^. *Y. lipolytica* wild type strains isolated are Generally Recognized As Safe (GRAS) from the American Food and Drug Administration (FDA) and Qualified Presumption of Safety (QPS) from European Food Safety Authority (EFSA)^4,8,9^. It is used as platform for the production of proteins (specially proteases and lipases)^2^ and organic acids (citric acid, isocitric acid, acetic acid)^2,9,10^.

Citric acid is the most widely used organic acid in the world with an annual production of the purified molecule estimated at 1.8 million tons^11^. This production is carried out mainly by microbial fermentation of diverse carbon sources (glucose, glycerol and *n*-alkanes)^12^. The mold *Aspergillus niger* is practically the only microorganism used at industrial scale with nearly 90% of the total production^9,11^. However, the direct use of the *A. niger* on a food matrix is not feasible since it is not GRAS and presents a high risk of contamination.

The completion of the *Y. lipolytica* genome and genome-scale models (GEM) allowed investigations of the cells physiology and cellular metabolism involved in citric acid secretion and intracellular lipid accumulation^13–19^. Production of extracellular citric acid or intracellular lipid is triggered by the imbalance between carbon sources in excess and limited biogenic substrates [nitrogen (N), iron (Fe), inorganic phosphate (Pi) and zinc (Zn)]^9,12,20^. The mostly used parameter to trigger this metabolic shift is a N-limitation (N-lim) in culture medium, allowing a high C/N ratio thus resulting in citrate secretion from an overflow metabolism at high glucose concentration^21^. Isocitrate dehydrogenase (IDH), a reversible Krebs enzyme involved in isocitrate to α-ketoglutarate conversion, is reported as key enzyme for lipid accumulation and citrate production in oleaginous microorganisms^22–24^. Authors proposed that upon N-lim, adenosine monophosphate (AMP) deaminase enzyme catalyze AMP to IMP and NH3 conversion to compensate for nitrogen depletion. Lower intracellular AMP leads to IDH inhibition and both isocitrate and citrate accumulation. Then citrate and isocitrate are excreted to extracellular medium but citrate can also be converted to acetyl-CoA, used as precursor for lipid synthesis^25,26^.

Recent studies highlighted the key role of oxygen on citrate optimized production in *Y. lipolytica* cultures^27–29^. Indeed, it has been showed that increased oxygen mass transfer rate (OTR) improves up to threefold citrate production by *Y. lipolytica* W29 strain^28^. Similarly, recent investigations led to citrate optimization by controlling the dissolved oxygen concentration (pO_2_)^27,29^. Finally, in this older work, authors reported that citrate overproduction is inversely correlated to the cyanide-resistance respiratory pathway on *Candida/Yarrowia lipolytica*^30^. Cyanide resistance pathway is mainly present in plants and fungi and involves an alternative oxidase (AOX) protein^31,32^; along with II NADH dehydrogenase (NDH2e), both part of the uncoupled ΔΨ-independent mitochondrial respiration^33,34^. The exact mechanism involving AOX on citrate production is not clearly determined but NDH2e-AOX pathway has been proposed as defense mechanism against Reactive Oxygen Species (ROS) accumulation in *Y. lipolytica* at stationary phase^35^. Although mitochondria are the main sources of ROS, other cytoplasmic proteins have recently been characterized as sources of ROS. This is the case of the long chain fatty alcohol oxidase (FAO). FAOs are located in the endoplasmic reticulum and participate in the oxidation of long chain alkanes or fatty acids, and production of long-chain aldehyde and hydrogen peroxide (H_2_O_2_) They have been mainly studied in industrial yeasts *Candida tropicalis* or *Candida cloacae*^36^ and recently identified in *Yarrowia lipolytica*^37^.

When glucose is used as a sole carbon source, citric acid production by *Y. lipolytica* at stationary phase is favored over lipid accumulation^38^. Recent studies have implemented strategies for controlling substrate feeding rate for lipid accumulation preventing the production of citric acid^21^. The authors managed to identify a range of N/C ratios favoring the production of lipid without citric acid excretion. Similarly, a GEM of *Y. lipolytica* has been proposed in combination with dynamic Flux Balance Analysis (dFBA), a computational algorithm, to study environmental conditions favorable for lipid accumulation over citric acid production^13^. These authors found that the cell specific oxygen consumption rate (rO_2_) is reduced during lipid accumulation. Applying a fed-batch strategy under reduced dissolved oxygen concentration, from 50 to 1%, they improved lipid accumulation, from 200 mg/gDCW to 400 mg/gDCW and avoid citrate excretion. Computational strategies such as dFBA allow to analyze genome-scale reconstructed metabolic networks and to run knock-out or environment-based optimization strategies for biomass of metabolites production^19,39,40^.

FBA predicts flux balances based on constraints and objective functions for biochemical networks verifying steady-state assumption^39,41^. The mathematical description of the metabolic reactions relies on the stoichiometries of reactants and products and does not require enzyme kinetics parameters. Dynamic flux balance analysis (dFBA) extends the FBA framework to help model how metabolic states change over time and interact with their environment. The method was developed by Varma and Palsson to predict the behavior of *E. coli* during several batch runs^42^. It was then formalized by Mahadevan et al. who presented two different approaches to dynamic flux balance analysis, called the dynamic optimization approach (DOA) and the static optimization approach (SOA)^43^. The DOA method optimizes over the entire time period of interest to obtain time profiles of fluxes and metabolite levels using non-linear programming problem. The SOA method divides time in discrete intervals and a new FBA problem is solved at time *t* after updating the external conditions according to the FBA solution at time *t* − 1.

Five GEMs are known from the literature so far. The *iNL895* model was the first genome-wide metabolic network of *Y. lipolytica*^15^. This model is based on the old consensus model of *Saccharomyces cerevisiae*, to which was added the lipid network. The *iYL619_PCP* model was released the same year and experimentally validated by studying growth on glucose and mineral medium^16^. This GEM was updated with more detailed reactions and became *iYL_2.0*. It has been used to optimize the accumulation of triacylglycerol (TAG)^18^. Similarly, the model *iMK735* was developed to study conditions for lipid optimization in *Y. lipolytica*^13^. This model was validated with dFBA on mineral medium with glucose or glycerol as sole carbon source. The *iYali4* model was constructed from the consensus network of *S. cerevisiae*^44^, *iNL895* and *iYL619_PCP*. The model has been applied to the study of amino acid metabolism and the overflow phenomenon associated to lipid accumulation^17^. We consider that *iYali4* model is the most complete regarding lipid metabolism, compared to previous models, and has been updated based on the genome sequence of the strain *Y. lipolytica* W29 used in this study^45^.

Here, we used the *Yali4* GEM and dFBA SOA strategies with COBRA Toolbox under MATLAB to analyze flux distributions associated with citrate production and alternative oxidase (AOX) protein activity. For this end, we adjusted the *iYali4* model with the mitochondrial AOX reaction identified in *Y. lipolytica*^46,47^ and performed dFBA to identify metabolic pathways contributing to citrate optimization, using *gurobi* solver (Gurobi Optimizer) for linear programming (LP).

## Methods

### Microorganism and media composition

The *Yarrowia lipolytica* W29 wild-type strain was used in this study. It is from CIRM-Levures collection and was provided by Micalis, INRA (Jouy-en-Josas, France) and stored at − 80 °C on 50% (v/v) glycerol.

Two media were used in this study. The YPD medium is composed of 10 g/L yeast extract, 10 g/L peptone (BD Biosciences, France) and 20 g/L glucose [Merck (Sigma-Aldrich), USA]. The mineral medium [Merck (Sigma-Aldrich), USA] was adapted from Ochoa-Estopier et al. (2014) and Moeller et al. (2007) with the following components (g/L): 1 (NH4)_2_SO_4_; 6 KH_2_PO_4_; 1.5 MgSO_4_·7H_2_O; 0.02 CuSO_4_-5H_2_O; 0.01 MnSO_4_·H_2_O; 0.0105 ZnCl_2_; 0.0035 FeSO_4_·7H_2_O; CaCl_2_·2H_2_O; 0.00025 D-Biotin; 0.001 D-L-Panthotenic acid, 0.001 Nicotinic Acid; 0.00625 Myo-Inositol; 0.001 Thiamin, 0.001 Pyridoxine; 0.0002 para-Aminobenzoic Acid. Glucose was used as carbon source and added to a final concentration of 50 g/L.

### Culture conditions

All pre-cultures were cultivated in 200 mL Erlenmeyer flasks on YPD medium at pH 6 and incubated on a rotary shaker at 28 °C and 100 rpm. Culture broth was taken at exponential growth phase, after 16–18 h of cultivation, centrifuged and washed in a 9 g/L NaCl prior to inoculation in mineral medium for both bioreactors and shaked flasks culture.

Bioreactor culture (n = 1) were performed in the 7 L Applikon system. The temperature was maintained at 28 °C and pH at 6 by the addition of 5 M NaOH. Dissolved oxygen was maintained above 20% from air saturation by controling the stirrer speed between 300–600 rpm and aeration at 2 vvm of air and dioxygen from 0 to 1 vvm. An antifoaming agent (B200 K) was added to 0.09% v/v at inoculation.

Culture in Erlenmeyer flask (n = 1) were performed at the same condition as the pre-culture, on a rotary shaker at 28 °C. The pH of the culture was maintained between 5 and 6 by manually and periodically adding 300 µL of 5 M NaOH in sterile condition.

40 mM *n*-propyl Gallate (nPG) stock solution were prepared on 70% ethanol and periodically added to cultures, to a final 400 µM concentration. It is a well-known inhibitor of the alternative oxidase (AOX) enzyme and already used to study mitochondrial respiration in *Yarrowia lipolytica*^35^.

### Biomass and metabolites analysis

Cell growth was followed by a spectrophotometer with calculation of yeast optical density (OD) at 600 nm. Dried biomass was carried out and calibrated against the DO_600 nm_ for the calculation of extracellular metabolic fluxes per gram of dry cell weight (gDCW). We found 0.62 gDCW/L concentration of biomass with DO_600 nm_ = 1, for *Y. lipolytica* W29 grown on mineral media.

For the determination of extracellular concentrations, 5 mL of the fermentation broth was centrifuged at 5×*g* for 5 min, aliquoted in 1 mL microtubes (Eppendorf, Germany) and stored at − 20 °C until further analysis. 500 µL Nanosep centrifugal tubes (VWR, USA) were used to filter the supernatant prior to metabolites quantification by High Performance Liquid Chromatography (HPLC) using ion chromatography system (Thermo Scientific ICS 5000+). IonPac AS11-2 × 250 mm column (Thermo Scientific, USA), IonPac AG11-2 × 500 mm pre-column (Thermo Scientific, USA) and AERS 500, 2 mm (Thermo Scientific, USA) were used for organic acids (citrate, isocitrate, succinate, acetate) quantification and with the following conditions: 35 °C column temperature, 0.5 mL/min NaOH (100 mM) eluant flow with gradient concentrations. Glucose concentration was determined with the Dionex CarboPac PA1 column 4 × 50 mm (Thermo Scientific) equipped with amperometric detection. Residual ammonium (NH4^+^) was quantified by a colorimetric essay (#1080240001, Merck (Sigma-Aldrich), USA).

### Need for accurate *Y. lipolytica* GEM description

**Figure 1:**
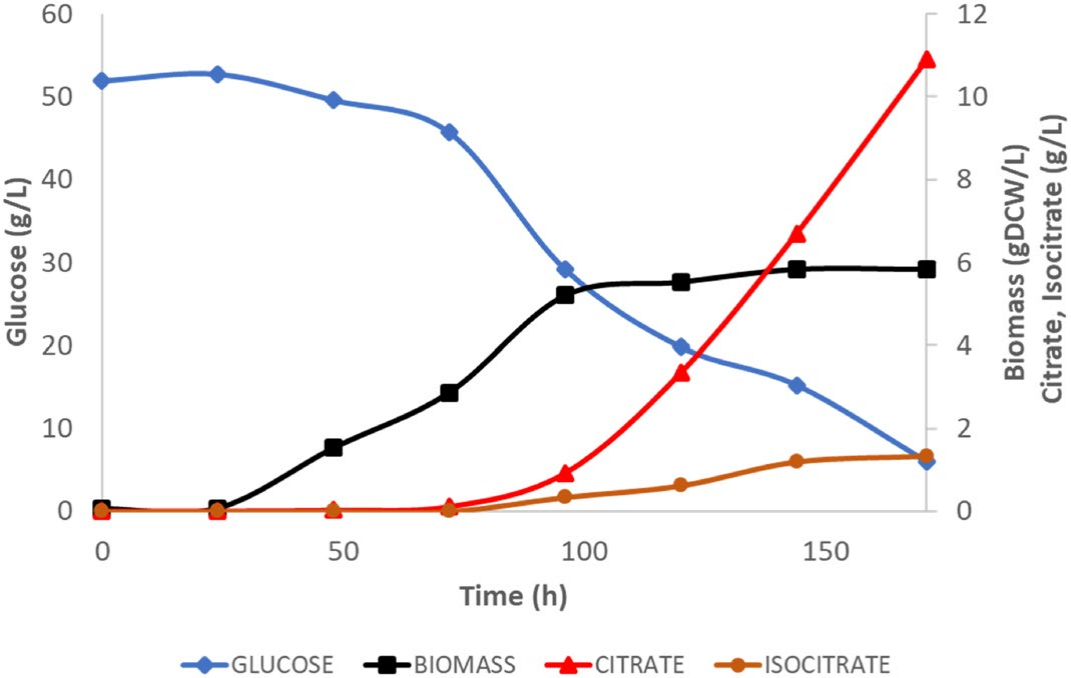
Experimental data obtained from batch (n = 1) of *Y. lipolytica* culture. Citrate and isocitrate are produced upon cultures reach stationary phase.

We used *iYali4* the GEM of *Y. lipolytica* by Kerkhoven et al., and an updated version for *Y. lipolytica* W29 to study citrate optimization. Growth rate on glucose and nitrogen-limited mineral medium was predicted by the model based on glucose specific consumption rate adequately fitted to the experimental results (Table 1 and Fig. 1). It was noted that *iYali4* and the other GEMs did not integrate the alternative oxidase (AOX) reaction. So we added the AOX reaction as part of the electron transfer chain (ETC), based on the hypothesis that this enzyme has a protective effect against ROS generation in *Y. lipolytica* and probably on citrate production^30,31,35^. The stoichiometry of AOX reaction were determined from literature and in coherence with *iYali4* ETC reaction: ubiquinol + 0.25 02 =  > ubiquinone + 0.5 H_2_O^32^. AOX reaction is doesn’t contribute to proton pumping across the mitochondrial membrane^48^.

**Table 1:** Kinetic physiological parameters.

Initial glusoce conc. (g/L)	100 g/L	50 g/L	Simulation
Growth rate (h^−1^)	0.076 ± 0.004	0.049 ± 0.004	0.052
q_Gluc_ (mmol/gDCW/h)	0.34 ± 0.027	1.148 ± 0.264	1.148
q_O2_ (mmol/gDCW/h)	0.6	1.1	1.1
r_Cit_ (mmol/gDCW/h)	0.301	0.093	0.093
r_Icit_ (mmol/gDCW/h)	0.140	0.060	0.060

## Results

### Experimentally determined kinetic parameters

We performed a series of bioreactor batch cultivations of *Yarrowia lipolytica* in mineral medium for kinetic parameters determination. These cultures were running in nitrogen-limited (N-lim) conditions to favor citrate production. Glucose was used as carbon source. Initial two glucose concentrations were accessed in order to determine the N/C ratio effect on cells specific growth rate (*µmax*) and other kinetic physiological parameters (Table 1). Specific growth rate of 0.076 ± 0.004 h^−1^ is observed in the lower N/C ratio compared to 0.049 ± 0.004 h^−1^ in the higher N/C ratio condition. The opposite effect of the N/C ratio is observed for glucose uptake where higher glucose uptake of 1.148 ± 0.264 mmol/gDCW/h is observed for the higher N/C ratio condition. Citrate, isocitrate, pyruvate, acetate and succinate were detected in the batch cultures but only after extracellular nitrogen source (NH4^+^) is totally consumed (between 28 and 30 h of culture). Citrate and isocitrate specific production rates are higher under low N/C ratio batch cultivation. For the remaining of our investigations, we considered the higher N/C ratio condition to avoid the glucose inhibition effect at 100 g/L.

### In silico growth prediction

We first used FBA to challenge *Yali4* GEM accuracy on predicting *Y. lipolytica* growth. The experimentally determined kinetic parameters such as specific glucose and oxygen uptake at the higher N/C (0.00204) condition were used as constrains to predict cell growth behavior. The model predicts a growth rate of 0.052 h^−1^, with low 0.616% error (Table 1). Thus, the *Yali4* model accurately predicts the *Yarrowia lipolytica* W29 strain growth in mineral medium. However, this model applied to FBA algorithm was not able to predict the glucose inhibition effect on 100 g/L glucose concentration medium. We also tried the *iMK735* model, but it failed to predict accurate growth.

### Predicting citrate production upon N-lim

Nitrogen limitation (N-lim) is the mostly used limitation parameter to trigger lipid accumulation and citrate production by *Y. lipolytica*. Initial NH4^+^ and glucose concentrations were set to 102 mg/L and 50 g/L, respectively. Experimental data obtained from duplicate flasks show two growth phases (Fig. 1). In the first growth phase an exponential growth rate *µmax*_1_ = 0.100 ± 0.008 h^−1^ was determined between 45 and 50 h (only residual NH4^+^ remain in the culture medium and were qualitatively measure by colorimetric essay). A second stable growth phase, with a *µmax*_*2*_ of 0.0240 ± 0.0013 h^−1^ is observed thereafter until the culture reached stationary phase, at 96–100 h. Up to 6 gDCW/L of biomass concentration has been observed. Citrate production clearly started upon nitrogen starvation and cell growth limitation. It’s mainly produced during the cell growth stationary phase and reached 10.9 g/L at the end of cell cultivation. Lower isocitrate concentration of 1.3 g/L was then observed. After 170 h of culture, residual 5 g/L of glucose remained in the medium (Fig. 1).

**Figure 2:**
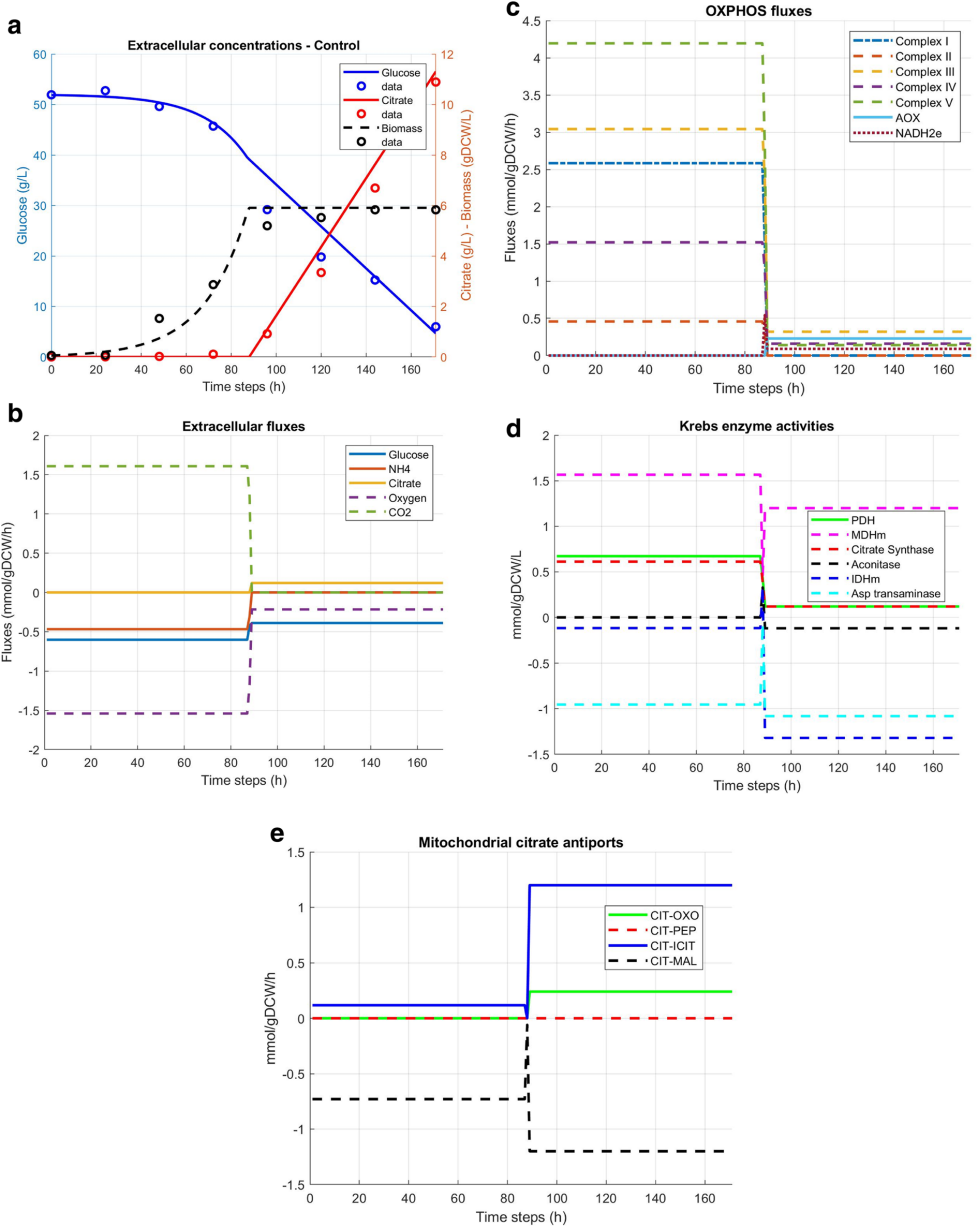
(**a**) Calibrating dFBA model with experimental flux rates allowed predictions close to experimental results, except for isocitrate which is not predicted by the model. (**b**) Simulated extracellular fluxes. Upon NH4^+^ limitation between 80 and 90 h, citrate flux increases. Glucose and oxygen consumption fluxes are lower during stationary phase (stat-phase). No CO_2_ production is predicted at stat-phase. (**c**) Simulated oxidative phosphorylation (OXPHOS) activity. OXPHOS activity is globally reduced upon stat-phase. AOX and NADH2e are inhibited on growth phase and activated during stat-phase. Complexes I is inhibited on stat-phase, which is a marker of a the metabolic switch, with limited energy demand and activation of the alternative respiration through NADH2e-AOX axes. (**d**) Characterization of Krebs enzymes activities. On growth phase, Krebs cycle is active since pyruvate dehydrogenase (PDH), citrate synthase (CS), aconitate 1 and 2, mitochondrial isocitrate dehydrogenase (ISDHm) and malate dehydrogenase (MDHm) have positive fluxes. During stat-phase carbon fluxes are redirected towards mitochondrial citrate accumulation. (**e**) Mitochondrial citrate antiports. Citrate-phosphoenolpyruvate (CIT-PEP) is inactive on both phases. Citrate-oxoglutarate (CIT-OXO) is active during stat-phase only. Both citrate-isocitrate (CIT-ICIT) and citrate-malate (CIT-MAL) increase their fluxes from growth to stat-phase.

The initial biomass concentration was set to 0.06 gDCW/L based on experimental data. Then we applied the dynamic Flux Balance Analysis (dFBA) algorithm to *Yali4* model with the aim to simulate our experimental results. Experimental initial glucose concentration (50 g/L) and uptake rate (0.64 mmol/gDCW/h) were first set in the model according to the experimental data. Slightly adjusting the specific glucose uptake rate down to 0.60 mmol/gDCW/h allowed to better fit the experimental curve. The alternative oxidase (AOX) reaction flux was fixed to zero during the growth phase, as reported by Guerrero-Castillo et al., and free-bounded during the stationary phase^35,49,50^. Therefore, a modified *dynamicFBA.m* MATLAB function has been proposed to allow further dFBA simulations when growth rate reaches the stationary phase (*µ* = 0). Then, the initial objective function of biomass optimization during the growth phase was changed to “lipid accumulation” as it’s experimentally observed upon stationary phase. Therefore, our dFBA model permitted prediction of biomass and citrate production in good agreement with the experimental results (Fig. 2a). Nevertheless, isocitrate production cannot be predicted by the model. The predicted specific growth rate of 0.052 h^−1^ differs from the experimental *µmax*_*1*_ (0.100 ± 0.008 h^−1^) but looks closer if we consider the average of the whole culture, i.e. the mean *µ* of the two successive growth phases (0.062 h^−1^).

### Reduced simulated OxPhos during citrate production and lipid accumulation

Glucose consumption reached the upper-bound rate value of 0.60 mmol/gDCW/h during exponential growth phase (exp-phase), and it was constrained to 0.39 mmol/gDCW/h at stationary growth phase (stat-phase). Predicted NH4^+^ and oxygen specific consumption rates are respectively 0.468 mmol/gDCW/h and 1.54 mmol/gDCW/h at exp-phase, and both drop to 0 and 0.217 mmol/gDCW/h respectively at stat-phase. Carbon dioxide (CO_2_) is produced only on exp-phase (Fig. 2b). These results are hallmarks of reduced cell energy demand. To specifically illustrate that, we investigated the simulated mitochondrial activity, emphasing the fluxes along the complexes of the electron transfer chain (ETC). The model reports reduced ATP production rate by complex V (CV) at stat-phase (Fig. 2c). Complex I (CI) and complex II (CII) are inhibited at stat-phase. The model predicts active complexes III (CIII) and complex IV (IV) on stat-phase with a drastic decrease. Further, the added alternative oxidase (AOX) reaction is active on stat-phase and reached 0.022 mmol/gDCW/h oxygen consumption rate. Surprisingly, the model also predicts NADH dehydrogenase (NADH2e) activity at stat-phase (0.08 mmol/gDCW/h) whereas this enzyme shows being inactive during exp-phase. This is a hallmark of the uncoupled mitochondrial respiratory pathway as reported by Guerrero-Castillo et al. NADH2e, instead of CI, supplies electrons to AOX through ubiquinone/ubiquinol cycling without contributing to the mitochondria intermembrane proton gradient. This result clearly suggests that this uncoupled alternative pathway is contributing to citrate production, as mainly observed on the stationary phase. We have then assessed this hypothesis proceeding by stimulating Krebs enzymes activity.

### Krebs enzymes activity sustain mitochondrial citrate accumulation at stat-phase

When the simulated growth reaches stat-phase, Krebs enzymes activity seem to sustain mitochondrial citrate accumulation. Pyruvate dehydrogenase (PDH) activity is decreased from 0.672 mmol/gDCW/h at exp-phase to 0.120 mmol/gDCW/h at stat-phase. It remains the dominant mitochondrial Acetyl-CoA supplier, joined with mitochondrial malate dehydrogenase which furnishes oxaloacetate for citrate synthesis (Fig. 2d). Citrate synthase (CS) activity also decreases from 0.612 to 0.120 mmol/gDCW/h. The model predicts inactive aconitase during stat-phase but active, on the reverse way, from isocitrate to citrate, during the stationnary growth phase. NAD-dependent mitochondrial isocitrate dehydrogenase (IDHm) activity is also reversely operating converting oxoglutarate to isocitrate. The cytosolic NADP-dependent counterpart (IDH) is inversely active during growth phases. Both isozymes show increased fluxes during stat-phase associated with citrate production. All the other Krebs enzymes, from oxoglutarate dehydrogenase (OXDH) to fumarase, are active during growth phase and totally inactive on stat-phase (Fig. 2d). Amino acids seem also involved in citrate accumulation since aspartate transamination is a source of oxaloacetate used by citrate synthase for mitochondrial citrate synthesis.

We also investigated mitochondrial-to-cytosolic citrate transport to decipher which pump is solicitated during growth and citrate production phases. Four mitochondrial membrane citrate antiports are identified in the *Yali4* model: citrate-phosphoenolpyruvate (CIT-PEP), citrate-oxoglutarate (CIT-OXO), citrate-isocitrate (CIT-ICIT) and citrate-malate (CIT-MAL). The model predicts inactive CIT-PEP on both phases. During growth phase, the model predicts mitochondrial to cytosolic citrate fluxes through CIT-MAL transporter and a lower inverse CIT-ICIT flux contributing to cytosolic isocitrate. During stat-phase, the net cytosolic citrate flux is ensured by CIT-OXO antiport since the positive CIT-MAL flux is totally reversed to mitochondria through CIT-ICIT antiport (Fig. 2e).

### Optimization of citrate synthesis

**Figure 3:**
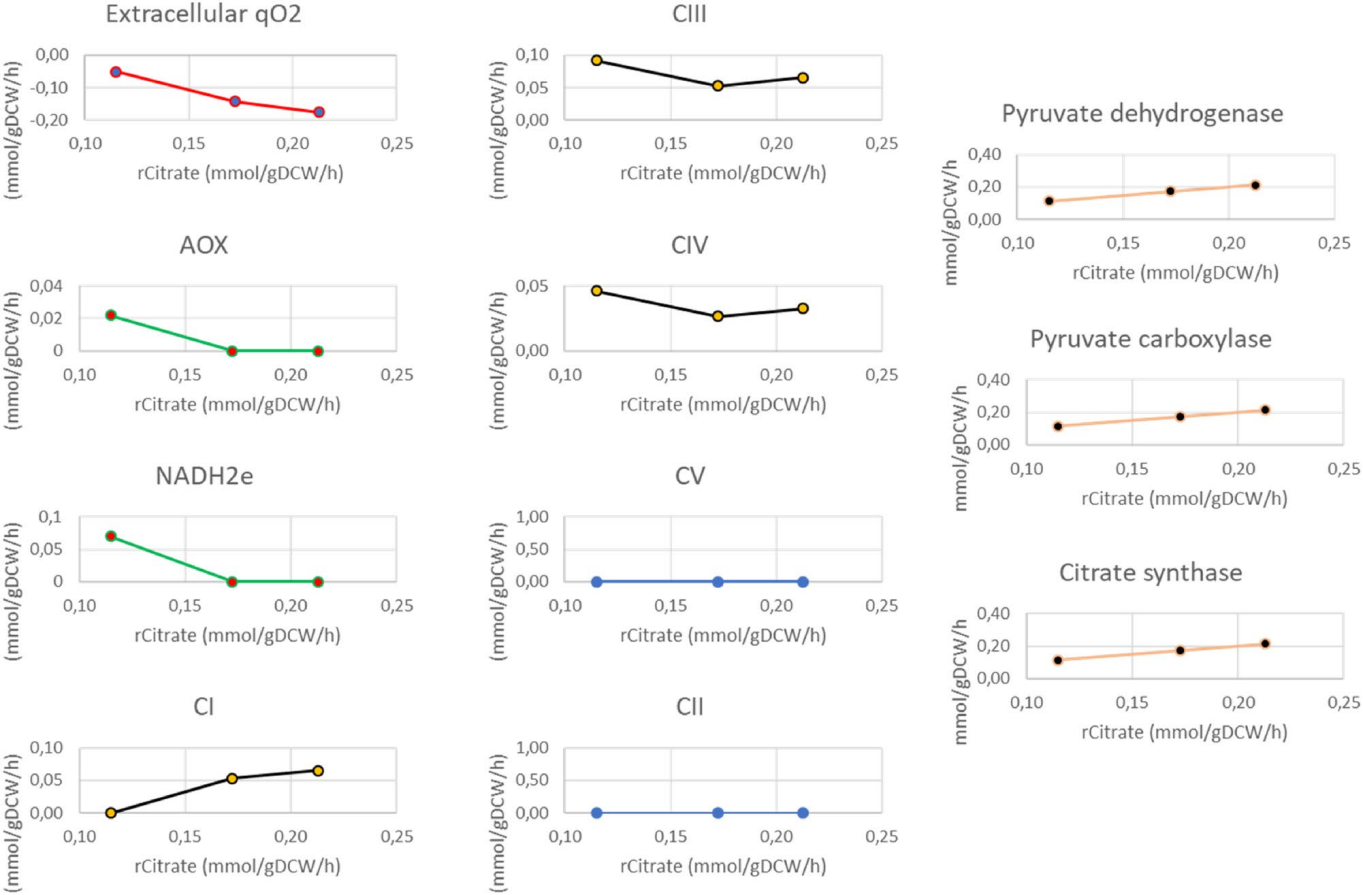
Simulated OXPHOS and enzymes activities at stationary phase with increased citrate production rate. Optimizing citrate production rate is correlated with limited oxygen consumption rate, decreased AOX and NADH2e. Complex I (CI) activity increases for NADH turnover. PDH, PC and CS supports citrate overproduction.

**Figure 4:**
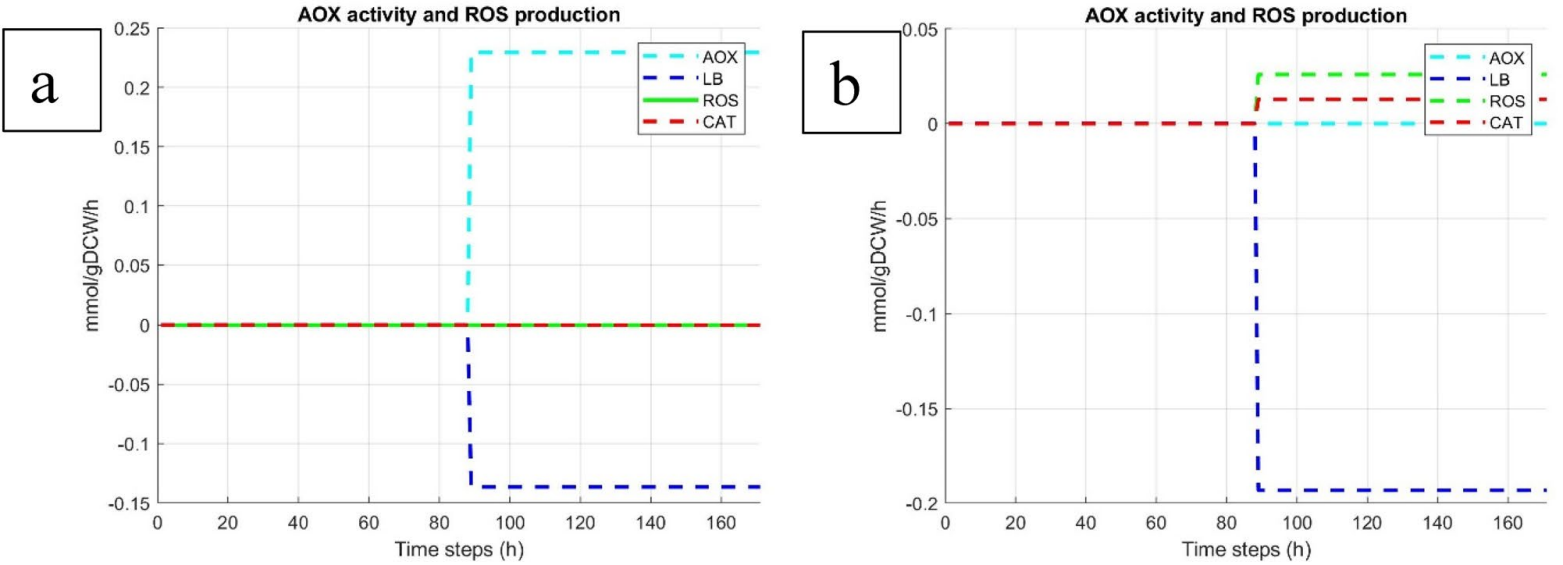
Simulated AOX and reactive oxygen species (ROS) production in normal condition (**a**) and in condition of optimized citrate production (**b**). Lipid bodies (LB) and ROS accumulation, catalase (CAT) activity increase on stat-phase concomitant with optimized citrate production. Fatty acid and alcohol oxidases contribute to ROS generation. On another hand catalase participates in cleaning ROS species.

**Figure 5:**
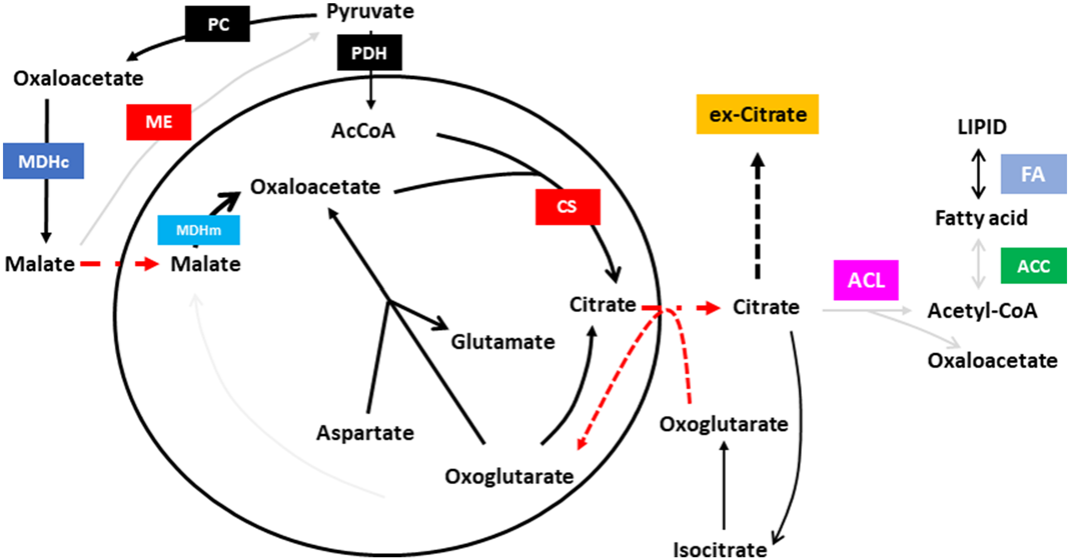
Illustrated balance of citrate overflow. Oxoglutarate and aspartate, malate and pyruvate mainly contribute to mitochondrial citrate overflow. ATP citrate lyase (ACL) inactivation allows cytosolic citrate accumulation and citrate production. Fluxes to lipogenesis is globally limited. Fatty acid accumulation from lipolysis is predicted to trigger ROS accumulation.

**Figure 6:**
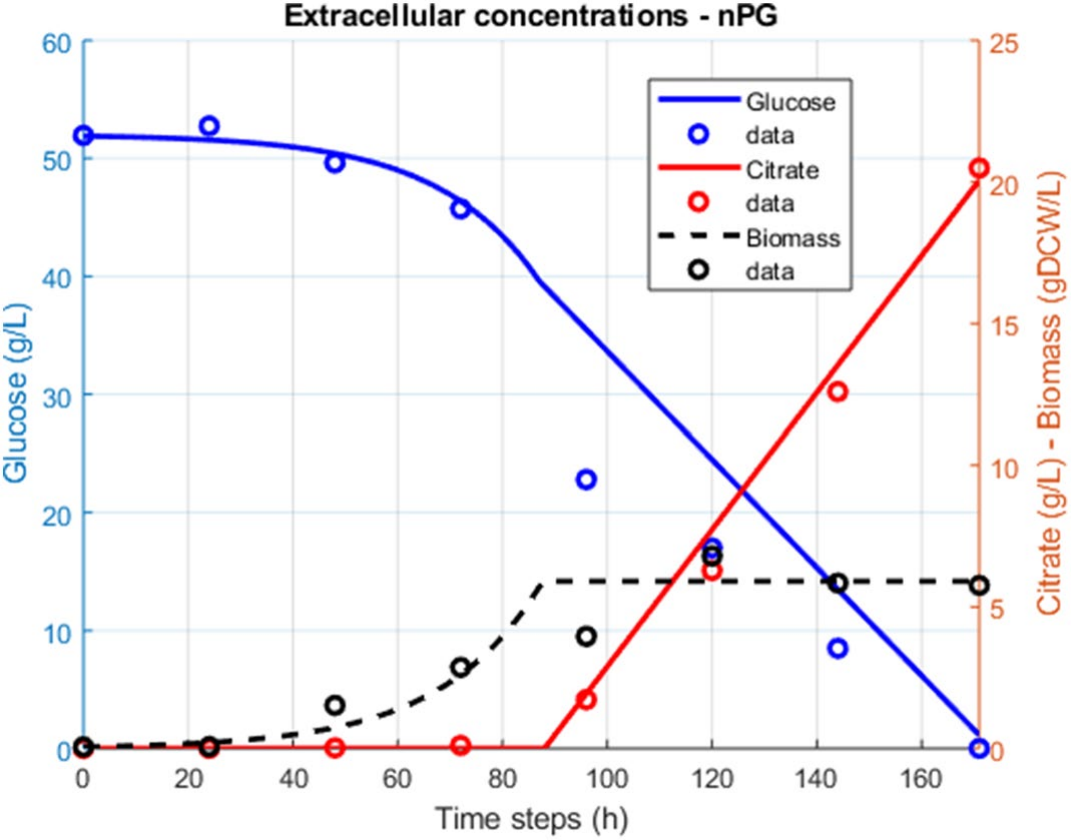
Inhibition of AOX activities by nPG triggers citrate production experimentally (n = 1) and in silico. Up to 20 g/L of citrate is produced during the stat-phase.

We also intended to investigate metabolic triggers promoting citrate overproduction. For that we simulated higher citrate production rate at stationary phase and identified major effectors (inhibiting or increasing reaction fluxes) of carbon overflow resulting in increased extracellular citrate production. We first restricted our study to Krebs enzymes, ETC complexes and reactions involved in the citrate pathway. Our results show that PDH-PC-CS route is the main contributor of carbon overflow to mitochondrial citrate accumulation. These simulated enzymes activities are proportional to citrate production rate (Fig. 3). Likewise, the mitochondrial aconitase works on the reversed direction and triggers isocitrate to citrate flux. A third phenotype associated with citrate overproduction is an increased extracellular oxygen consumption rate (Fig. 3). This is counterintuitive since less ATP is required during the stationary growth phase and OxPhos complexes activities are decreased, except for the complex I (CI), which increases concomitantly with citrate production rate. Interestingly, the simulated increased CI flux is not accompanied with overexpressed complexes III and IV. Looking more closely at the intracellular reactions, it appears that cytosolic reaction of long-chain alcohol oxidation, ensured by fatty alcohol oxidase (FAO), is the main route of ROS production (Figs. 4, 5). Intracellular oxygen consumption is balanced by catalase reduction of hydrogen peroxidase (H_2_O_2_) with a specific 0.013 mmol/gDCW/h flux rate. This result also emphasizes that the NADH2e-AOX alternative route for NADH oxidation and O_2_ reduction is inactive when citrate is overproduced during the stationary phase (Figs. 4, 6). Based on these observations, we have then experimentally assessed the effect of AOX inhibition on citrate production in batch cultures of *Y. lipolytica* in shake flasks in mineral medium at 50 g/L of glucose. When the culture reached the stationary phase, we added 100 µM *n*-propyl gallate (nPG), a well-known inhibitor of AOX, at 72 h then repeated every 24 h. Experimental results show optimized citrate production in the nPG-implemented medium (Fig. 6). Up to 20.5 g/L of citrate is produced, compared to 10.9 g/L found on control media (Fig. 1), biomass reaches 5.8 gDCW/L and only traces of glucose remain at the end of the culture (Fig. 6). Experimental data are well predicted by the model, except for glucose consumption during stat-phase. To our knowledge this is the first report of *Y. lipolytica* citrate overproduction on mineral medium based on a culture management strategy designed using mathematical predictions.

Another way to confirm these predictions would have been to add salicylhydroxamic acid (SHAM), another well-known and used inhibitor of AOX^51^. The dFBA model of *Y. lipolytica* also was also used to predict the effect of adding various Krebs intermediates such as α-ketoglutarate, malate and oxaloacetate for citrate optimization. Whereas simulations reported an improved citrate production (data not showed), these results weren’t experimentally confirmed, compared to the control (Supplementary Fig. SI II).

## Discussion

Few studies already reported optimization of citrate in *Aspergillus niger* and *Yarrowia lipolytica* by modulating mitochondrial metabolism with specific effector compounds or by cell engineering strategies^52,53^. Our predicted results show that the oxidative phosphorylation (OxPhos) pathway is globally reduced when the cell culture reaches the stationary phase (Fig. 2c). These results were confirmed by a series of oxygraphic experiments (cell respiration) carried out at different growth states, therefore with cells exhibiting different mitochondrial membrane potential (Supplementary Fig. SI. I). We observed that mitochondrial respiration is functional though the alternative NADH2e-AOX pathway whereas complex I (CI) is inactive (Fig. 2c). This is in good agreement with the proposed association/dissociation mechanism of NADH2e/CIV channeling electron from the external NADH to cytochrome pathway or towards the alternative oxidase^35^. In agreement with the literature we propose AOX activity as a protective mechanism against mitochondrial ROS production in *Y. lipolytica* and some other yeasts and plants species^51,54^. Indeed, OxPhos is considered as the main ROS producer, specially through CI and CIII^54^. Our results of simulated intracellular oxygen consumption upon citrate optimization show that fatty acid oxidation may also be a source of ROS production. The *long-chain alcohol oxidase (C12)* reaction provided by the fatty alcohol oxidase gene (FAO) consumes O_2_ at stationary phase and triggers the oxidation of cytosolic alcohols. Theses reaction is accompanied by the production of hydrogen peroxide (H_2_O_2_) as major ROS species. FAO gene has been recently identified in *Y. lipolytica* as key in ω-oxidation of long-chain fatty acids^37^. One may consider a global mechanism in *Y. lipolytica* where citrate is mainly used for lipids accumulation at the entry in stationary phase, followed by ω and β-oxidation of fatty acids upon lipid saturation and exposure to free oxygen species. Fatty acids oxidation is thus thought to trigger cell defense mechanisms such as catalase (CAT) and alternative NADH2e-AOX oxidase to circumvent ROS accumulation (Fig. 4). Further oxygenation of the culture by controlling pO_2_ or OTR or inhibition of AOX activity is then expected as a key to trigger citrate overproduction. The proposed mechanism is in good agreement with the work of Kavšček et al., and Sabra et al., showing that the fine modulation of oxygen consumption enzymes in *Y. lipolytica* favors citrate overflow metabolism.

A recent study of citrate production in *Aspergillus niger* by disruption or overexpression of *aox1* gene, in addition to antimycin A during cell growth, showed an improved citrate production in overexpressed *aox1* strains compared to the parental strain^55^. Up to 169.1 g/L citrate is reported for one of the overexpressed strains against 158.9 g/L for the parental stain. According to our understanding of the results presented, the highest amount of citrate obtained for the overexpressed *aox1* strain should only be attributed to a better germination rate. Moreover, antimycin A compound used in the study is stated as an inhibitor of AOX protein, which is in contradiction with the effect sought by the overexpression of *aox1*. In the absence of contradicting our study, Hou et al. (2018) confirm the hypothesis that AOX may contribute to clearing ROS species and improving cell survival.

## Conclusion

We applied dFBA algorithm to the existing *Yali4* model of *Yarrowia lipolytica* W29 strain metabolism to predict favorable metabolic conditions for citrate optimization. The model first predicted carbon overflow to lipid accumulation and citrate production during stationary phase. *Y. lipolytica* respiration is drastically decreased during citrate production phase and shifts from OxPhos to the alternative ΔΨ-independent pathway ensured by NADH2e/AOX route. Isocitrate dehydrogenase and aconitase are predicted to operate in the backward direction and favor mitochondrial citrate accumulation. Simulated overproduction of citrate is accompanied by inhibition of the NADH2e/AOX pathway as well as increased lipid oxidation and ROS production. The prediction of citrate overproduction has been confirmed experimentally by inhibition of AOX protein. We also challenged the importance of ROS in balancing lipid accumulation and citrate production fluxes by adding glutathione to *Y. lipolytica* culture in the presence of nPG. Our results showed a global inhibition of citrate production. We propose AOX gene as a target for metabolic engineering and industrial production of citrate in food and pharmaceutical industries.

## Supplementary Information

Supplementary Information.

## References

[1] Barth, G. & Gaillardin, C. *Yarrowia lipolytica*. In *Nonconventional Yeasts in Biotechnology* 313–388 (Springer, Berlin, 1996). 10.1007/978-3-642-79856-6_10.

[2] Nicaud J-M (2012). *Yarrowia lipolytica*. Yeast.

[3] Lazar Z, Liu N, Stephanopoulos G (2018). Holistic approaches in lipid production by *Yarrowia lipolytica*. Trends Biotechnol..

[4] Groenewald M (2014). *Yarrowia lipolytica*: Safety assessment of an oleaginous yeast with a great industrial potential. Crit. Rev. Microbiol..

[5] Dulermo T, Nicaud J-M (2011). Involvement of the G3P shuttle and β-oxidation pathway in the control of TAG synthesis and lipid accumulation in *Yarrowia lipolytica*. Metab. Eng..

[6] Papanikolaou S, Aggelis G (2003). Selective uptake of fatty acids by the yeast *Yarrowia lipolytica*. Eur. J. Lipid Sci. Technol..

[7] Blazeck J (2014). Harnessing *Yarrowia lipolytica* lipogenesis to create a platform for lipid and biofuel production. Nat. Commun..

[8] Ricci A (2018). Update of the list of QPS‐recommended biological agents intentionally added to food or feed as notified to EFSA 8: Suitability of taxonomic units notified to EFSA until March 2018. EFSA J..

[9] Anastassiadis S, Morgunov IG, Kamzolova SV, Finogenova TV (2008). Citric acid production patent review. Recent Pat. Biotechnol..

[10] Papanikolaou S, Muniglia L, Chevalot I, Aggelis G, Marc I (2002). *Yarrowia lipolytica* as a potential producer of citric acid from raw glycerol. J. Appl. Microbiol..

[11] Sawant O, Mahale S, Ramchandran V, Nagaraj G, Bankar A (2018). Fungal citric acid production using waste materials: A mini-review. J. Microbiol. Biotechnol. Food Sci..

[12] Max B (2010). Biotechnological production of citric acid. Braz. J. Microbiol..

[13] Kavšček M, Bhutada G, Madl T, Natter K (2015). Optimization of lipid production with a genome-scale model of *Yarrowia lipolytica*. BMC Syst. Biol..

[14] Dujon B (2004). Genome evolution in yeasts. Nature.

[15] Loira N, Dulermo T, Nicaud J-M, Sherman DJ (2012). A genome-scale metabolic model of the lipid-accumulating yeast *Yarrowia lipolytica*. BMC Syst. Biol..

[16] Pan P, Hua Q (2012). Reconstruction and in silico analysis of metabolic network for an oleaginous yeast, *Yarrowia lipolytica*. PLoS ONE.

[17] Kerkhoven EJ, Pomraning KR, Baker SE, Nielsen J (2016). Regulation of amino-acid metabolism controls flux to lipid accumulation in *Yarrowia lipolytica*. NPJ Syst. Biol. Appl..

[18] Wei S, Jian X, Chen J, Zhang C, Hua Q (2017). Reconstruction of genome-scale metabolic model of *Yarrowia lipolytica* and its application in overproduction of triacylglycerol. Bioresour. Bioprocess..

[19] Mishra P (2018). Genome-scale model-driven strain design for dicarboxylic acid production in *Yarrowia lipolytica*. BMC Syst. Biol..

[20] Yalcin SK, Bozdemir MT, Ozbas ZY (2010). Citric acid production by yeasts: Fermentation conditions, process optimization and strain improvement. Curr. Res. Technol. Educ. Top. Appl. Microbiol. Microb. Biotechnol..

[21] Ochoa-Estopier A, Guillouet SE (2014). D-stat culture for studying the metabolic shifts from oxidative metabolism to lipid accumulation and citric acid production in *Yarrowia lipolytica*. J. Biotechnol..

[22] Evans CT, Ratledge C (1985). Possible regulatory roles of ATP:citrate lyase, malic enzyme, and AMP deaminase in lipid accumulation by *Rhodosporidium toruloides* CBS 14. Can. J. Microbiol..

[23] Ratledge C, Wynn JP (2002). The biochemistry and molecular biology of lipid accumulation in oleaginous microorganisms. Adv. Appl. Microbiol..

[24] Filipp FV, Scott DA, Ronai ZA, Osterman AL, Smith JW (2012). Reverse TCA cycle flux through isocitrate dehydrogenases 1 and 2 is required for lipogenesis in hypoxic melanoma cells. Pigment Cell Melanoma Res..

[25] Beopoulos A, Nicaud J-M, Gaillardin C (2011). An overview of lipid metabolism in yeasts and its impact on biotechnological processes. Appl. Microbiol. Biotechnol..

[26] Zhang H, Wu C, Wu Q, Dai J, Song Y (2016). Metabolic flux analysis of lipid biosynthesis in the yeast *Yarrowia lipolytica* using 13C-labled glucose and gas chromatography-mass spectrometry. PLoS ONE.

[27] Sabra W, Bommareddy RR, Maheshwari G, Papanikolaou S, Zeng A-P (2017). Substrates and oxygen dependent citric acid production by *Yarrowia lipolytica*: Insights through transcriptome and fluxome analyses. Microb. Cell Fact..

[28] Ferreira P, Lopes M, Mota M, Belo I (2016). Oxygen transfer rate and pH as major operating parameters of citric acid production from glycerol by *Yarrowia lipolytica* W29 and CBS 2073. Chem. Pap..

[29] Kamzolova SV, Shishkanova NV, Morgunov IG, Finogenova TV (2003). Oxygen requirements for growth and citric acid production of *Yarrowia lipolytica*. FEMS Yeast Res..

[30] Akimenko VK, Finogenova TV, Ermakova IT, Shishkanova NV (1979). Respiratory cyanide resistance in *Candida lipolytica* and the supersynthesis of citric acids. Mikrobiologiia.

[31] Akimenko VK, Arinbasarova AY, Smirnova NM, Medentsev AG (2003). The alternative oxidase of *Yarrowia lipolytica* mitochondria is unable to compete with the cytochrome pathway for electrons. Microbiology.

[32] Berthold DA, Andersson ME, Nordlund P (2000). New insight into the structure and function of the alternative oxidase. Biochim. Biophys. Acta BBA Bioenergy.

[33] Kerscher SJ (2000). Diversity and origin of alternative NADH:ubiquinone oxidoreductases. Biochim. Biophys. Acta BBA Bioenergy.

[34] Joseph-Horne T, Babij J, Wood PM, Hollomon D, Sessions RB (2000). New sequence data enable modelling of the fungal alternative oxidase and explain an absence of regulation by pyruvate. FEBS Lett..

[35] Guerrero-Castillo S, Cabrera-Orefice A, Vázquez-Acevedo M, González-Halphen D, Uribe-Carvajal S (2012). During the stationary growth phase, *Yarrowia lipolytica* prevents the overproduction of reactive oxygen species by activating an uncoupled mitochondrial respiratory pathway. Biochim. Biophys. Acta.

[36] Cheng Q (2005). Candida yeast long chain fatty alcohol oxidase is a c-type haemoprotein and plays an important role in long chain fatty acid metabolism. Biochim. Biophys. Acta BBA Mol. Cell Biol. Lipids.

[37] Gatter M (2014). A newly identified fatty alcohol oxidase gene is mainly responsible for the oxidation of long-chain ω-hydroxy fatty acids in *Yarrowia lipolytica*. FEMS Yeast Res..

[38] Papanikolaou S (2009). Biosynthesis of lipids and organic acids by *Yarrowia lipolytica* strains cultivated on glucose. Eur. J. Lipid Sci. Technol..

[39] Orth JD, Thiele I, Palsson BØ (2010). What is flux balance analysis?. Nat. Biotechnol..

[40] Raman K, Chandra N (2009). Flux balance analysis of biological systems: Applications and challenges. Brief. Bioinform..

[41] Varma A, Palsson BO (1994). Metabolic flux balancing: Basic concepts, scientific and practical use. Nat. Biotechnol..

[42] Varma A, Palsson BO (1994). Stoichiometric flux balance models quantitatively predict growth and metabolic by-product secretion in wild-type *Escherichia coli* W3110. Appl. Environ. Microbiol..

[43] Mahadevan R, Edwards JS, Doyle FJ (2002). Dynamic flux balance analysis of diauxic growth in *Escherichia coli*. Biophys. J..

[44] Aung HW, Henry SA, Walker LP (2013). Revising the representation of fatty acid, glycerolipid, and glycerophospholipid metabolism in the consensus model of yeast metabolism. Ind. Biotechnol. New Rochelle N.

[45] Magnan C (2016). Sequence assembly of *Yarrowia lipolytica* strain W29/CLIB89 shows transposable element diversity. PLoS ONE.

[46] Joseph-Horne T, Hollomon DW, Wood PM (2001). Fungal respiration: A fusion of standard and alternative components. Biochim. Biophys. Acta BBA Bioenergy.

[47] Kerscher S, Dröse S, Zwicker K, Zickermann V, Brandt U (2002). *Yarrowia lipolytica*, a yeast genetic system to study mitochondrial complex I. Biochim. Biophys. Acta.

[48] Vishwakarma A, Tetali SD, Selinski J, Scheibe R, Padmasree K (2015). Importance of the alternative oxidase (AOX) pathway in regulating cellular redox and ROS homeostasis to optimize photosynthesis during restriction of the cytochrome oxidase pathway in *Arabidopsis thaliana*. Ann. Bot..

[49] Guerrero-Castillo S (2011). Physiological uncoupling of mitochondrial oxidative phosphorylation. Studies in different yeast species. J. Bioenergy Biomembr..

[50] Guerrero-Castillo S, Vázquez-Acevedo M, González-Halphen D, Uribe-Carvajal S (2009). In *Yarrowia lipolytica* mitochondria, the alternative NADH dehydrogenase interacts specifically with the cytochrome complexes of the classic respiratory pathway. Biochim. Biophys. Acta.

[51] Garcia-Neto W, Cabrera-Orefice A, Uribe-Carvajal S, Kowaltowski AJ, Alberto Luévano-Martínez L (2017). High osmolarity environments activate the mitochondrial alternative oxidase in *Debaryomyces Hansenii*. PLoS ONE.

[52] Wang L (2015). Inhibition of oxidative phosphorylation for enhancing citric acid production by *Aspergillus niger*. Microb. Cell Fact..

[53] Papanikolaou S (2013). Importance of the methyl-citrate cycle on glycerol metabolism in the yeast *Yarrowia lipolytica*. J. Biotechnol..

[54] Vanlerberghe GC (2013). Alternative oxidase: A mitochondrial respiratory pathway to maintain metabolic and signaling homeostasis during abiotic and biotic stress in plants. Int. J. Mol. Sci..

[55] Hou L (2018). Functional analysis of the mitochondrial alternative oxidase gene (aox1) from *Aspergillus niger* CGMCC 10142 and its effects on citric acid production. Appl. Microbiol. Biotechnol..

