# Supplementary material for: Fine-tuning mitochondrial activity in Yarrowia lipolytica for citrate overproduction
Source: Sci Rep. 2021 Jan 13;11:878. doi: 10.1038/s41598-020-79577-4 (PMC7807019; doi:10.1038/s41598-020-79577-4)
Supplement: Supplementary file 1 — Supplementary Information. [file 41598_2020_79577_MOESM1_ESM.docx]

# **Supplementary material**

**Fine-tuning mitochondrial activity in Yarrowia lipolytica**

**for citrate overproduction**

**Jorgelindo da Veiga Moreira^1^, Mario Jolicoeur^1^, Laurent Schwartz^4^ and Sabine Peres*^2,3^**

^1^Research Laboratory in Applied Metabolic Engineering, Department of Chemical Engineering, Ecole Polytechnique de Montréal, P.O. Box 6079, Centre-Ville Station, Montréal (Quebec), Canada

^2^LRI, Université Paris-Sud, CNRS, Université Paris-Saclay, 91405 Orsay, France

^3^MaIAGE, INRA, Université Paris-Saclay, 78350 Jouy-en-Josas, France

^4^Assistance Publique des Hôpitaux de Paris, 149 avenue Victoria 75004 Paris, France

***Corresponding author:** speres@lri.fr


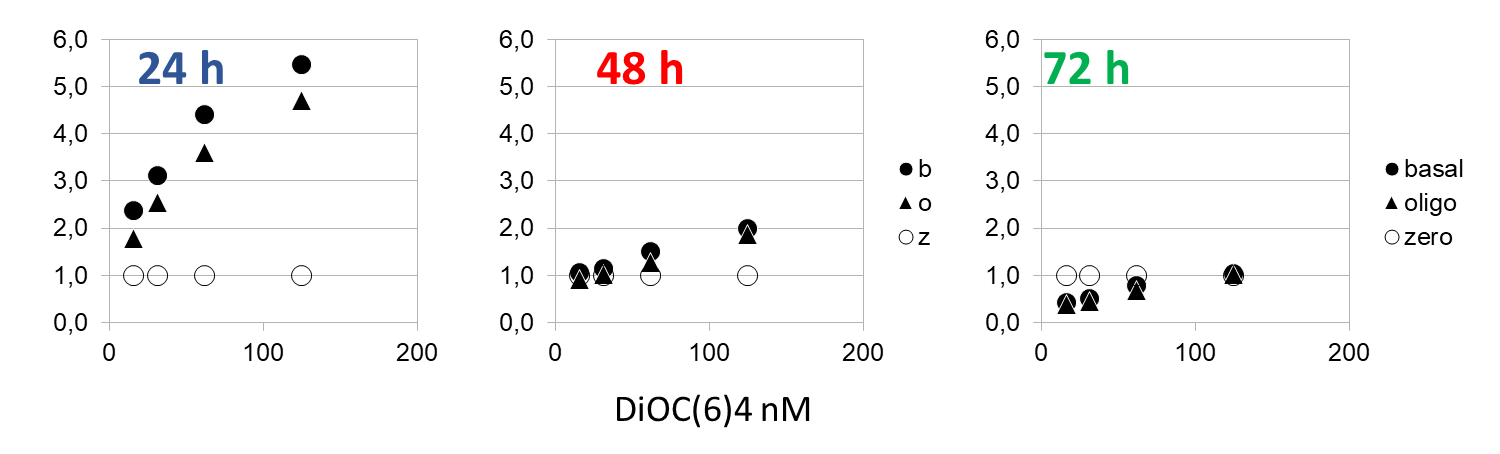


Figure SI 1: Experimental measurements of mitochondrial membrane potential (MMP) at 24h, 48h and 72h of *Y. lipolytica* culture. DiOC(6) marker were used at different concentrations to evaluate MMP. Basal curves are MMP without OXPHOS inhibition. Oligo condition are MMP reported with oligomycin addition to cell culture. MMP are expressed on fold-change compared to ‘zero’ condition where all complexes’ inhibitors were added to the culture. MMP globally decreases with culture time and practically inhibited on stat-phase (72h).


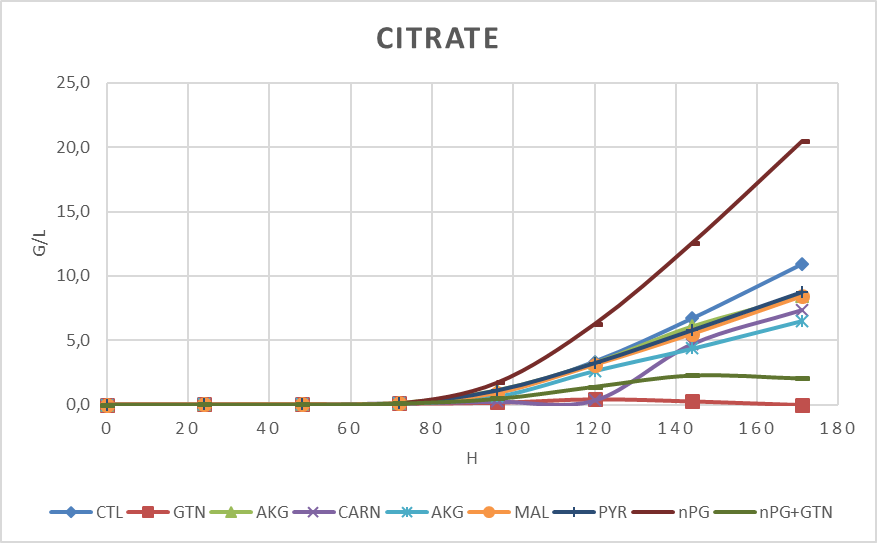


Figure SI 2: Experimental results of citrate production with addition of deferent metabolites on stat-phase. Compared to the control (CTL) condition, nPG addition stimulates citrate overproduction whereas other Krebs metabolites, α-ketoglutarate/oxoglutarate (AKG), carnitine (CARN), malate (MAL), pyruvate (PYR), do not significantly change citrate production. Glutathione (GTN) addition to *Y. lipolytica* culture vanishes citrate production. When nPG is added jointed to GTN, citrate is produced but to a lesser extent.
